# Expression of hybrid fusion protein (Cry1Ac::ASAL) in transgenic rice plants imparts resistance against multiple insect pests

**DOI:** 10.1038/s41598-018-26881-9

**Published:** 2018-05-31

**Authors:** Dayakar Boddupally, Srinath Tamirisa, Sivakrishna Rao Gundra, Dashavantha Reddy Vudem, Venkateswara Rao Khareedu

**Affiliations:** Centre for Plant Molecular Biology, Osmania University, Hyderabad, 500007 India

## Abstract

To evolve rice varieties resistant to different groups of insect pests a fusion gene, comprising DI and DII domains of *Bt* Cry1Ac and carbohydrate binding domain of garlic lectin (ASAL), was constructed. Transgenic rice lines were generated and evaluated to assess the efficacy of Cry1Ac::ASAL fusion protein against three major pests, viz., yellow stem borer (YSB), leaf folder (LF) and brown planthopper (BPH). Molecular analyses of transgenic plants revealed stable integration and expression of the fusion gene. *In planta* insect bioassays on transgenics disclosed enhanced levels of resistance compared to the control plants. High insect mortality of YSB, LF and BPH was observed on transgenics compared to that of control plants. Furthermore, honeydew assays revealed significant decreases in the feeding ability of BPH on transgenic plants as compared to the controls. Ligand blot analysis, using BPH insects fed on *cry1Ac::asal* transgenic rice plants, revealed a modified receptor protein-binding pattern owing to its ability to bind to additional receptors in insects. The overall results authenticate that Cry1Ac::ASAL protein is endowed with remarkable entomotoxic effects against major lepidopteran and hemipteran insects. As such, the fusion gene appears promising and can be introduced into various other crops to control multiple insect pests.

## Introduction

Rice (*Oryza sativa* L.) is one of the major food grain crops which serves as a staple diet for more than two-thirds of the world’s population. Annually, about 24–41% of rice yield is lost due to diverse pests and diseases^1^, resulting from the widespread infestation of insect pests with rare feeding habits. Among the major insects of rice belonging to hemiptera (sap-sucking planthoppers) and lepidoptera (stem borers and leaf folders) are difficult to control and manage under field conditions. Amongst lepidopteran insects, yellow stem borer (YSB) (*Scirphophaga incertulus* Walker), striped stem borer (SSB) (*Chilo supressalis* Walker) and leaf folder (LF) (*Cnaphalocrosis medinalis* Guenee) proved to be major pests causing significant yield losses of up to 10–30% each year^2^. Brown planthopper (BPH) is one of the most notorious rice pest among hemipteran insects, which feeds mainly on stems and assimilates the phloem sap, causing wilted tillers and withered leaves. Further, BPH also serves as vector for transmitting viruses leading to severe decline in the rice productivity^3^.

Insecticidal Cry proteins from the bacterium *Bacillus thuringiensis* (*Bt*) are widely used as topical sprays besides their deployment in transgenic plants for insect control. These toxins have been expressed in various crops like maize, potato, tomato, cotton, brinjal, and rice for controlling major insect pests. However, they were found effective only against lepidopteran, coleopteran, and dipteran insect pests but were ineffective against hemipteran pests^4–6^. The current understanding of the mode of action of Cry toxins indicates that *Bt* inclusions get solubilized in the digestive tract of target insects, Cry protoxins are then activated and subsequently bind to the receptors on the epithelium of insect midgut and lyse the cells^7^. The toxic core structure of Cry1Ac (DI-II-III) is a soluble monomeric protein with Domain I being involved in toxin oligomerization, membrane insertion as well as pore formation^8^; while Domain II is associated with binding to specific larval midgut proteins and Domain III has a functional role in receptor recognition^9^. Strategies adopted for alteration of *Bt* toxin binding affinity and specificity can be divided into four classes, viz., domain or loop swapping between Cry toxins, site-directed mutagenesis, incorporation of binding peptides or fragments from non-Bt toxins, as well as generation and subsequent display of Cry toxin mutant libraries on phage^10^.

The proteins of plant origin such as lectins have been found effective against different sap-sucking insects. Lectins recognize and preferentially bind to carbohydrate complexes protruding from glycolipids and glycoproteins^11^. These carbohydrate-binding proteins contain two or more binding sites per subunit which can reversibly bind to specific sugar segments. In addition, they are involved in defense against phytopathogenic microorganisms, phytophagous insects and plant eating animals. The insecticidal activity of plant lectins against a large array of insect species belonging to several orders have been well documented^1^. Resistance to proteolytic degradation by the insect digestive enzymes and binding to insect gut membranes are the two important attributes that enable lectins to exert their deleterious effects on insects^12^. Garlic lectin ASAL binds to the carbohydrate part of the 55 kDa and 45 kDa brush border membrane vesicle receptor proteins in hemipteran insects. Binding of ASAL to these receptors decreases the permeability of the membrane and interfere with the digestive, protective or secretary functions of the intestine. Accordingly, lectins can adversely affect weight gain in the larvae, which in turn retard their development into pupae^13,14^.

In artificial diet assays, mannose-specific lectins revealed a significant anti-metabolic effect towards nymphs of the rice brown planthopper and a high mortality against the red cotton bug (*Dysdercus cingulatus*)^15,16^. The insecticidal activity of the ASAL expressed in transgenic cotton caused detrimental effects on larval development, besides growth and survival of the major lepidopteran pest *Spodoptera littoralis*^17^. Transgenic rice expressing ASAL exhibited explicit resistance against hemipteran insects BPH, green leafhopper (GLH) and white backed planthopper (WBPH)^1,18^. Similarly, transgenic cotton lines expressing ASAL displayed enhanced resistance against two major sap-sucking (jassid and whitefly) pests^19^.

The structural similarity between third domain of Cry1Ac and carbohydrate binding of ASAL lectin has been well studied^20^. In several cases, exchanging Domain III of the Cry1 toxins with other Cry1 toxins resulted in the enhanced toxicity of Cry proteins^21–24^. Previous studies indicated that Cry toxins, despite being activated in the gut of hemipteran insects, showed almost no toxicity^25,26^. In our earlier studies, based on the structural similarity between the receptor binding domain III of Cry and the carbohydrate binding domain of ASAL, the domain DIII was replaced with the carbohydrate binding domain of lectin and the resulting fusion-protein (Cry1Ac::ASAL) was evaluated for its toxic effects against major lepidoptern insects^20,27^. The overall results of *in silico* and *in vitro* studies on Cry1Ac::ASAL fusion protein revealed its higher binding energies and increased affinity towards insect receptors as compared to the parental proteins^20,27^.

In the present study, transgenic rice plants expressing Cry1Ac::ASAL fusion protein have been generated and tested against different insect pests using *in planta* bioassays. Transgenic rice plants exhibited significantly higher levels of resistance to three major insects of rice belonging to lepidopteran and hemipteran pests. Therefore, the engineered Bt toxin (fusion protein), which proved to be more effective against both the chewing (lepidopteran) and sucking (hemiptera) insects, holds great promise to meet the challenge of future pest management under the changed climatic conditions.

## Results

### Construction of a plant expression cassette containing *cry1Ac:*:*asal* fusion gene

The *cry1Ac::asal* hybrid gene was constructed by fusion of DI and DII domains of Cry1Ac with carbohydrate binding domain of ASAL. The fusion gene (*cry1Ac::asal*) was amplified and was cloned into the pBSSK (+) vector, and restriction analysis of the recombinant plasmid showed the presence of a 1464 bp fragment (Supplementary Fig. S1a). The fusion gene was further cloned into pRT100 vector and restriction analysis of the recombinant vector with *Nco* I & *Bam* HI enzymes confirmed the presence of 1089 bp and 375 bp fragments corresponding to *cry1Ac* and *asal* (Supplementary Fig. S1b). The *cry1Ac::asal* gene driven by CaMV35S promoter along with its *polyA* terminator was excised and cloned at the *Hind* III site of pCAMBIA3300 binary vector, which when digested with *Hind* III released a 2.2 kb band corresponding to the pCAMBIA3300*-cry1Ac::asal*-*bar* expression unit (Supplementary Fig. S1c).

### Generation of transgenic rice lines expressing the fusion gene (*cry1Ac:*:*asal*) and identification of stable transformants

**Figure 1:**
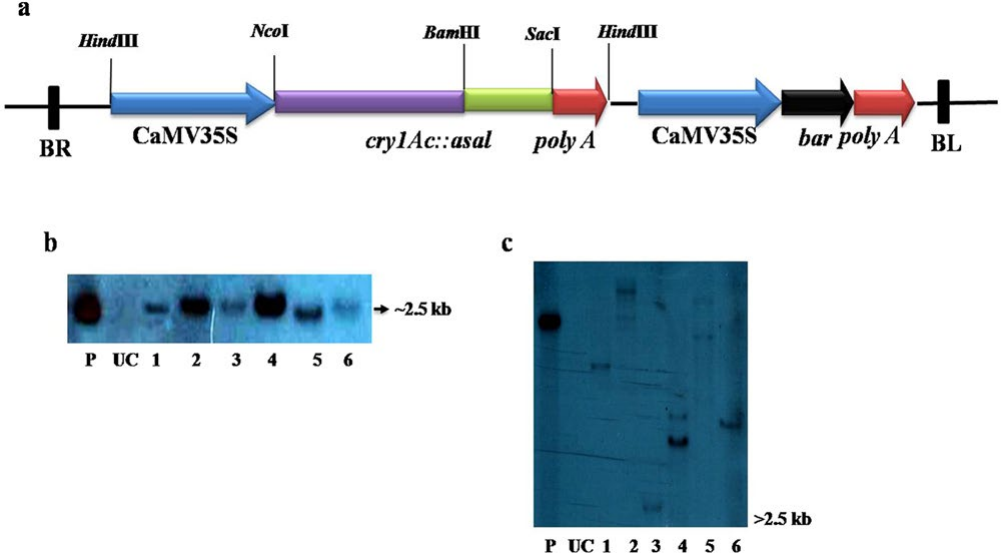
Restriction map of T-DNA region of pCAMBIA3300-*cry1Ac::asal–bar* and Southern blot analysis of transgenic plants. (**a**) Restriction map of T-DNA region containing CaMV 35S-*cry1Ac::asal* expression unit and *bar* selectable marker gene. (**b**) Genomic DNA digested with *Hind*III and probed with *cry1Ac::asal* coding sequence. (**c**) Genomic DNA digested with *Eco*RI and probed with *bar* coding sequence. Each lane was loaded with 15 µg of genomic DNA digested with respective enzymes. Lane P: Positive control (**b**) *cry1Ac::asal* expression unit and (**c**) *bar* expression unit. Lane UC: DNA from untransformed control plant. Lanes 1, 2, 3, 4, 5 & 6: DNA from PB-F_1D2_, PB-F_3D4_, PB-F_4D2_, PB-F_8D4_, PB-F_17D1_ and PB-F_20A5_ transgenic lines of Pusa Basmathi.

Embryogenic calli of Pusa Basmathi (PB1) were co-cultivated with the *Agrobacterium* strain EHA105 harbouring pCAMBIA3300*-cry1Ac::asal*-*bar* expression vector for the introduction of fusion gene into the rice genome (Fig. 1a). A total of twenty independent transgenic rice lines were generated from the selected calli grown on PPT (6–8 mg/l) containing medium. Among twenty transformants, six transgenic plants showed consistent high tolerance to the Basta (0.25%) herbicide in repeated tests (Supplementary Fig. S2a). PCR analyses of DNA extracts of Basta tolerant transgenic rice plants exhibited amplification of 1089 bp and 560 bp products corresponding to *cry1Ac* and *bar* transgenes, while no such bands were observed in the control plants (Supplementary Fig. S2b,c).

### Southern blot, RT-PCR and Western blot analyses of transgenic plants

Southern blot analysis of six transformants, viz., PB-F_1D_, PB-F_3D_, PB-F_4D_, PB-F_8D_, PB-F_17D_, and PB-F_20A_ when probed with the *cry1Ac::asal* coding sequence revealed a specific hybridizable band of ~2.5 kb (Fig. 1b). Further, when probed with the *bar* coding sequence hybridizable bands of varied sizes (>2.5 kb) were observed in different rice transformants (Fig. 1c). Whereas, untransformed control plants failed to show similar hybridization bands.

**Figure 2:**
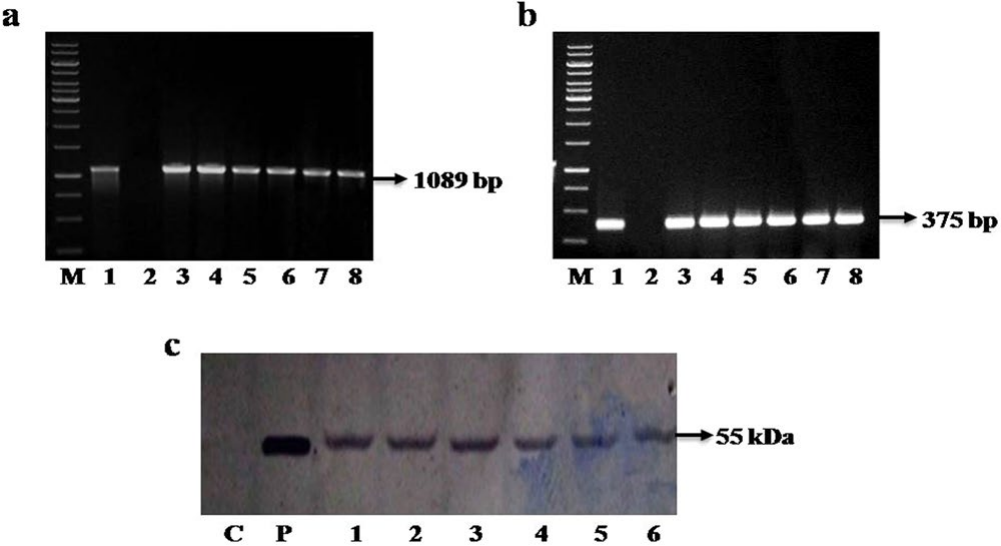
RT PCR and Western blot analyses of *cry1Ac::asal* transgenic rice plants along with control plants. (**a**) RT PCR analysis using RNA isolated form transgenic and control plants with primers corresponding to *cry1Ac* region. Amplified products were analyzed by agarose gel electrophoresis. Lane M: 1Kb DNA marker, Lane 1: Positive control, Lane 2: Untransformed control; Lanes 3–8: Different *cry1Ac::asal* transgenic rice plants. (**b**) RT PCR analysis using RNA isolated form transgenic and control plants with primers corresponding to *asal* region. Lane M: 1Kb DNA marker, Lane 1: Positive control, Lane 2: Untransformed control; Lanes 3–8: Different *cry1Ac::asal* transgenic rice plants. (**c**) Western blot analysis using the protein extracted from transgenic rice and control plants. Lane C: Protein extract (5 μg) from untransformed control plant, Lane P: Protein extract from purified protein; Lanes 1, 2, 3, 4, 5 & 6: Protein extracts (5 μg) from PB-F_1D2_, PB-F_3D4_, PB-F_4D2_, PB-F_8D4_, PB-F_17D1_, and PB-F_20A5_ transgenic lines.

RT-PCR analysis of Southern-positive transgenic plants revealed the presence of *cry1Ac::asal* gene transcripts as evidenced by the amplification of 1089 bp and 375 bp bands corresponding to *cry1Ac* and *asal* regions (Fig. 2a,b). Furthermore, different transgenic plants showed various intensities of both *cry* and *asal* transcripts. Western blot analyses of transgenic plants demonstrated the presence of ~55 kDa Cry1Ac::ASAL protein when treated with ASAL antibodies (Fig. 2c). Moreover, the amount of fusion protein quantified by ELISA analysis using Cry1Ac anti-body which revealed the presence of Cry1Ac::ASAL in varied amounts ranging from 1.0% to 1.8% in different transgenic plants.

### Evaluation of the entomotoxic effects of Cry1Ac::ASAL fusion protein on the lepidopteran insects of rice

**Figure 3:**
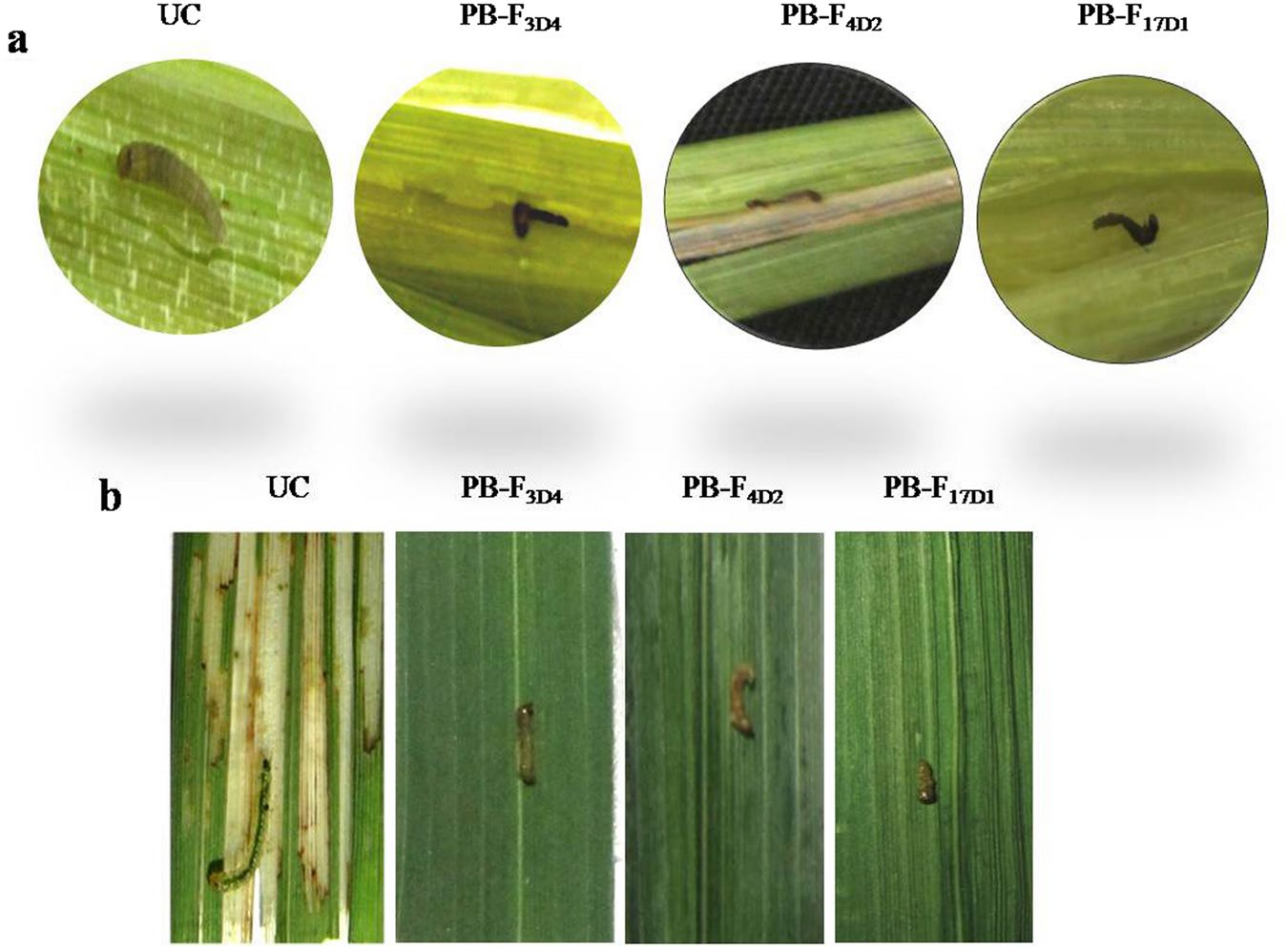
Bio-assay of transgenic rice plants against yellow stem borer and leaf folder. (**a**) Yellow stem borer insects fed on six weeks old untransformed control plant (UC) and PB-F_3D4,_ PB-F_4D2_ & PB-F_17D1_ transgenic plants. (**b**) Leaf folder insects fed on six weeks old untransformed control plant (UC) and PB-F_3D4,_ PB-F_4D2_ & PB-F_17D1_ transgenic plants.

For evaluation of entomotoxic toxic effects of Cry1Ac::ASAL fusion-protein expressed in the transgenic rice plants, insect bioassays were carried out using two major lepidopteran insects, YSB and LF. Insect bioassays on six different transgenic lines confirmed the efficacy of Cry1Ac::ASAL fusion protein against two major pests of rice. Complete (100%) insect mortality was observed against YSB which were fed on cut stems of transgenic lines at the tillering stage (Fig. 3a). Similarly, mortality of LF fed on two-week-old transgenic rice lines was found to be 80–100% (Fig. 3b). Insect mortality was observed from third day in four transgenic rice lines, while in the remaining two lines it was delayed by two more days. The insect mortality was evident by the typical symptom of Cry toxin activity where larvae turned black. The percentage of leaf damage caused by LF larvae on PB-F_4D2_, PB-F_3D4_, PB-F_17D1_ and PB-F_20A5_ transgenic rice plants was 10 ± 1, 12 ± 2, 12 ± 1 and 12 ± 1, and on PB-F_1D2_ and PB-F_8D4_ lines it was 27 ± 3 and 24 ± 2, respectively, compared to 100% damage observed on untransformed control plants (Fig. 3b).

### Evaluation of the fusion protein against BPH insects

**Figure 4:**
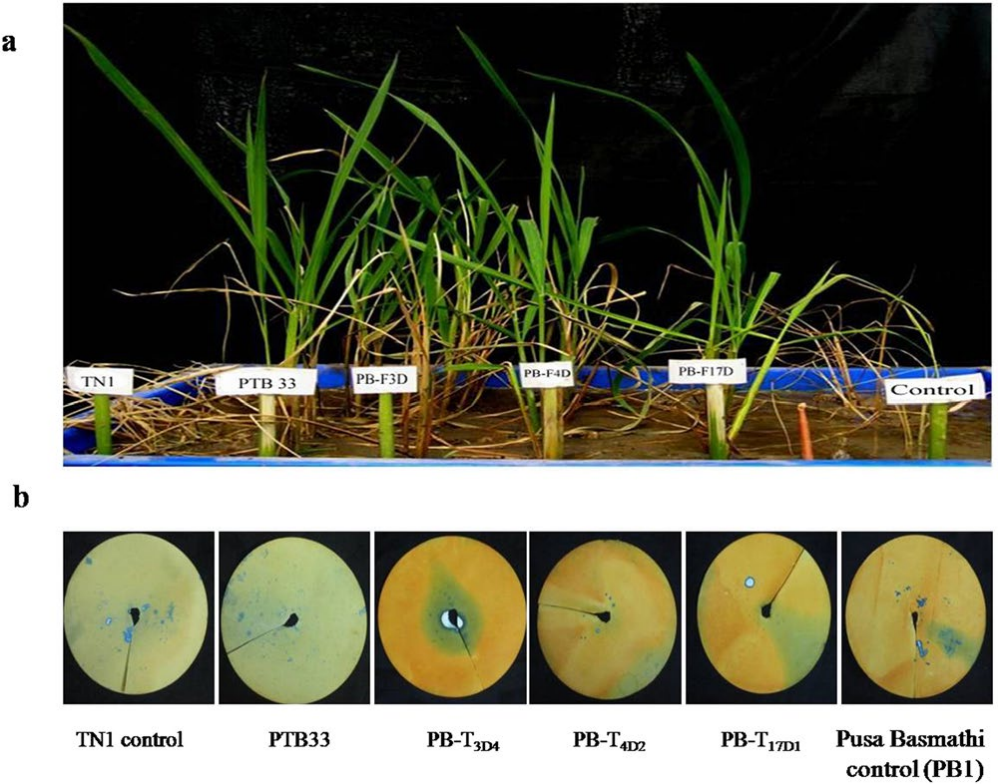
Evaluation of transgenic rice lines expressing Cry1Ac::ASAL fusion protein against brown planthopper (BPH). (**a**) Six weeks old transgenic lines along with respective controls infested with BPH nymphs. TN-1 and untransformed PB1 rice plants represent controls, var. PTB33 a Resistant check for BPH and PB-F_3D4_, PB-F_4D2_ and PB-F_17D1_ represent transgenic rice lines of PB1. Photographs were taken after 14 days of infestation. (**b**) Honeydew excretion by female BPH insects after 24 hours of feeding on controls and PB-F_3D4_, PB-F_4D2_ & PB-F_17D1_ transgenic rice plants.

**Figure 5:**
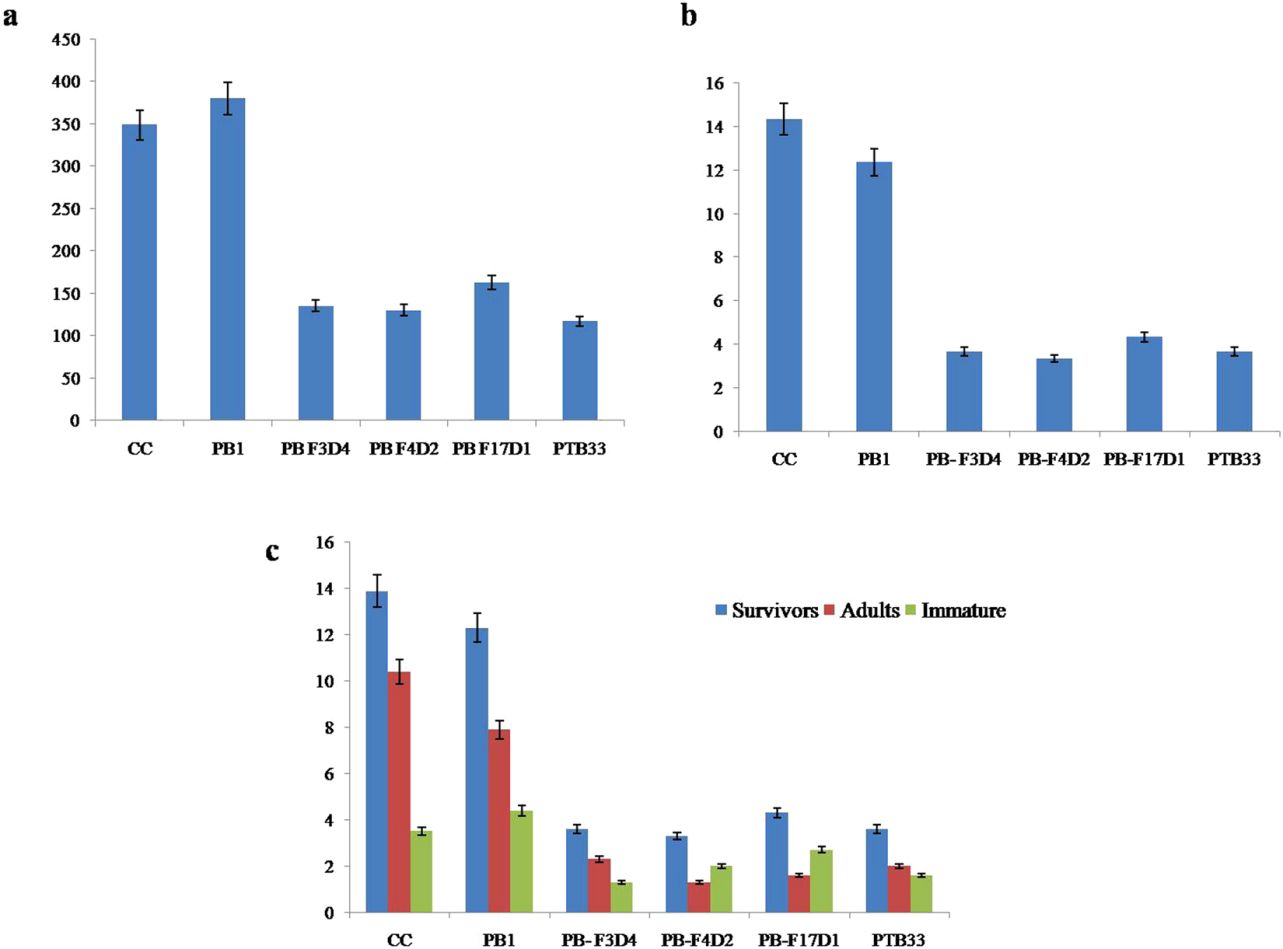
Survival, development, fecundity and feeding behaviour of BPH insects growing on transgenic rice lines expressing Cry1Ac::ASAL fusion protein. Twenty 1^st^ instar nymphs of BPH were released on each plant on day 0. Bioassays were carried out on 20 plants sampled from each transgenic line PB-F_3D4_, PB-F_4D2_ & PB-F_17D1_ and three controls TN1, PTB33, and untransformed PB1 (**a**) Survival rate of insects, (**b**) Insect development was recorded after 24 days. (**c**) Total number of nymphs produced by a pair of adult BPH insects on controls and transgenic plants were counted and the data was used to plot the graph. Bars indicate mean ± SE. (**d**) Feeding behaviour of BPH was estimated by honeydew excretion method and the data was used to plot the graph. Bars indicate mean ± SE.

Brown planthopper bioassays using *cry1Ac::asal* transgenic plants revealed significant mortality of larval nymphs and reduction in the fecundity of insects. Transgenic plants expressing Cry1Ac::ASAL fusion protein exhibited varied levels (1–3 score on a 0–9 scale) of resistance to BPH on par with those of BPH-resistant check var. PTB33 (Fig. 4a). Honeydew assay revealed significant reduction of ~ 87% to 93% in the feeding ability of BPH on transgenic plants when compared to the insects fed on the untransformed control plants (Fig. 4b). A mean number of 4.3 ± 0.27, 5.0 ± 0.47 and 10.0 **±** 0.48 honeydew units/plant were excreted by BPH, when fed on transgenic plants PB-F_4D2_, PB-F_3D4_ and PB-F_17D1_, respectively, compared with 124.0** ± **0.94 honeydew units/plant observed on the control plants (Fig. 5a). The survival of BPH on PB-F_4D2_, PB-F_3D4_ and PB-F_17D1_ transgenic rice plants was significantly reduced to 3.6 ± 1.4, 3.3 ± 1.6 and 4.3 ± 1.1 insects/plant, respectively, compared to 12.3 ± 2.9 insects/plant on untransformed control PB1 plants (Fig. 5b). Amongst survived BPH insects, only 20% to 30% could reach the adult stage on different transgenic lines as compared to insect survival on control plants (Fig. 5c).

### Ligand binding assays of BPH insects fed on transgenic rice

**Figure 6:**
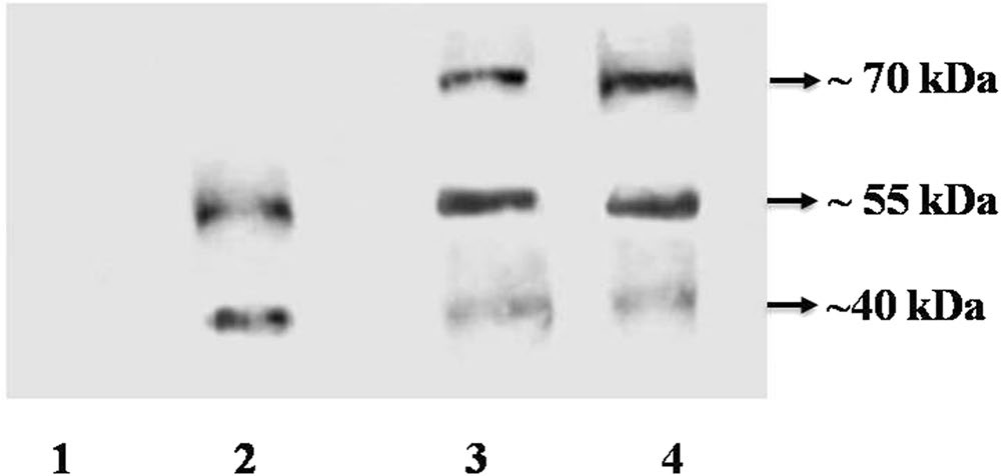
Ligand blot analyses of proteins extracted from BPH insects fed on transgenic and untransformed control plants. Protein was isolated from BPH insects and analysis was carried out using the ASAL antibodies. Lane 1: Protein extract (5 µg) from BPH insects fed on untransformed control plant; Lane 2: Protein extract (5 µg) from BPH insects fed on *asal*-transgenic plant; Lanes 3 and 4: Protein extracts (5 µg) from BPH insects fed on *cry1Ac::asal* transgenic rice lines.

Ligand binding assays were carried out to detect the BPH receptor proteins’ specificity to Cry1Ac::ASAL fusion protein. Proteins isolated from BPH insects fed on both transgenics (*asal*, and *cry1Ac::asal*) and control plants were treated with ASAL antibodies (Fig. 6). BPH insects fed on *cry1Ac-asal*-transgenic (PB-F_4D2_) plants disclosed three receptor proteins of ~70 kDa, ~55 kDa and ~40 kDa compared to ~55 kDa and ~40 kDa of insects fed on *asal*-transgenic plants developed in our previous study^1^. The bands observed on ligand blots represent complexes formed between the fusion protein and receptor proteins of the insects (Fig. 6). Whereas, BPH insects fed on control plants failed to show any detectable receptor protein specific to fusion protein (Fig. 6).

## Discussion

Globally, insects cause about 15% of direct losses to different agricultural crops as well as indirect losses owing to impaired quality of the produce^28^. Insects also act as vectors of various plant pathogens such as bacterial, fungal, and viral^29^. The threat of development of resistance by insect pests to a broad spectrum chemical insecticides has prompted research for adoption of alternative strategies. Several crop plants have been engineered with different *cry* genes conferring resistance against various major insect pests^30–32^. Rapid adoption and commercial introduction of *Bt* crops led to the development of resistance by insects against *Bt* toxins. Major pests, such as the diamondback moth, tobacco budworm, Colorado potato beetle, Indian mealy moth, maize stalk borer, cotton bollworm and fall armyworm have shown resistance to Cry toxins^33–37^. Moreover, Cry toxins are effective against lepidopterans but are ineffective against sucking pests belonging to hemiptera.

Plants, in general, are known to synthesize a wide range of defense proteins against different pathogens. The insecticidal activity of plant lectins against various insects belonging to hemipteran have been well documented^14^. Transgenic rice expressing ASAL exhibited ample resistance against sucking insects BPH, GLH and WBPH^1,18^. Pyramided transgenic rice lines containing *asal* and g*na* lectin genes exhibited enhanced resistance to major sap-sucking insects^38^. A number of successful fusion proteins were developed using lectin as a carrier protein. The observed increases in the mortality of insects caused by fusion proteins have been ascribed to the lectin domain, which enhanced the binding process and facilitated the entry of toxin more efficiently into the insect^39^. GNA when fused as a carrier protein for different chimeric toxins such as, Manase-AC/GNA^40^, SFlI/GNA^41^, Chitinase/GNA^42^, ButalT/GNA^43^ and ω-ACTX-Hv1a/GNA^44^ resulted in higher toxicities against various insect pests.

To overcome resistance acquired by insects against Cry toxins different strategies were employed to modify Cry functional domains to improve their toxicity^8,10^. Different gene fusions, viz., Cry1Ca, Cry1Fb, Cry1Ba modified with Cry1Ac domain III, Cry1Ac/ricin-B, Cry1Ac/CpTI, Cry1Ac/HWTX-I, Cry1Ac/CDEP2, and Cry1Ab/ACTX-Ar1, employed for engineering of plants bestowed with enhanced insect resistance^22,45–49^.

In the present study, transgenic plants expressing Cry1Ac::ASAL fusion protein have been generated and tested against different insect pests adopting *in planta* bioassays. The binary vector pCAMBIA3300-CaMV35S-*bar*, containing *cry1Ac::asal* expression unit driven by CaMV35S promoter, was introduced into rice by employing the *Agrobacterium*-mediated genetic transformation method. PCR analyses of Basta tolerant plants showed amplification of 1089 bp and 560 bp products corresponding to *cry1Ac* and *bar* transgenes testifying the presence of these genes in the genomes of different transgenic plants (Supplementary Fig. S2). Southern blot analysis of transformants, when probed with the *cry1Ac::asal* coding sequence, revealed a specific hybridizable band of ~2.5 kb confirming stable integration of the fusion gene in different rice transformants (Fig. 1b). Appearance of different hybridizable bands of ≥2.5 kb, corresponding to the *bar* expression cassette with the *Eco*RI digested genomic DNA, attest the single copy and independent nature of the transformants (Fig. 1c). These observations further suggest that the T-DNA is integrated into the rice genome as a single copy without any rearrangement. Moreover, the presence of single copy integration of transgenes facilitate their stable expression and predictable pattern of inheritance^50^.

RT-PCR analysis revealed the presence of *cry1Ac::asal* gene transcripts in the transgenic plants as evidenced by the amplification of 1089 bp and 375 bp fragments corresponding to *cry1Ac* and *asal* (Fig. 2a,b). Western blot analyses of transgenic plants, when treated with ASAL antibodies, demonstrated the presence of ~55 kDa Cry1Ac::ASAL protein (Fig. 2c). Furthermore, ELISA analysis revealed varied amounts (1.0–1.8%) of Cry1Ac::ASAL protein in different transgenic rice plants owing to insertion of the transgene at different transcriptionally active sites in the rice genome.

For evaluation of entomotoxic effects of Cry1Ac::ASAL fusion-protein expressed in the transgenic rice plants, insect bioassays have been carried out employing YSB, LF and BPH insects. Insect bioassays have been done on six different transgenic lines for YSB and LF. For further evaluation against BPH, three best rice lines have been used. The results revealed 100% mortality of the stem borers when fed on cut stems of transgenics at the tillering stage. Also, the mortality of leaf folders grown on two-week-old transgenic rice plants ranged from 80–100% (Fig. 3a,b). The ability of Cry1Ac::ASAL fusion protein to induce high mortality of lepidopteran pests testify that the domains I and II of Cry1Ac in conjunction with ASAL domain can cause high level insect toxicity. Several hybrid Cry proteins Cry1Ab-Cry1B, Cry1Ac-Cry1Ab, and Polh–Cry1Ac developed by domain swapping exhibited increased toxicities and broader activities when compared to the parental proteins^51–53^. Transgenic rice plants expressing Cry1Ab and Cry1Ac fusion protein showed high resistance against leaf folder and yellow stem borer insect pests without any reduced yields^54^. Rice yellow stem borer and leaf folder fed on Cry1Ab- and Cry1Ac-expressing plants showed varied insect mortality (10–100%)^55,56^. A chimeric *B*. *thuringiensis* toxin Cry2AX1 expressed in rice proved to be effective against certain major lepidopteran insect pests^57^.

BPH bioassays on transgenic rice resulted in a significant mortality of larval nymphs as well as reduction in the fecundity of insects. Transgenic plants expressing Cry1Ac::ASAL fusion protein exhibited varied levels (1–3 score on a 0–9 scale) of resistance to BPH, on par with those of BPH-resistant check var. PTB33. The survival of BPH on transgenic rice plants was significantly reduced compared to untransformed control plants (Fig. 5b). Among BPH insects survived, only 20–30% could reach the adult stage on different transgenic lines as compared to the control plants, which is similar to the earlier reports of ASAL-expressing transgenic rice plants^1^. Honeydew assay revealed significant reduction in the feeding ability of BPH on transgenic plants as compared to the insects fed on control plants (Fig. 5a). The overall results amply indicate the enhanced efficacy of Cry1Ac::ASAL fusion protein against the three major insect pests of rice.

The ligand binding assays on BPH insects fed on the transgenic rice plants expressing ASAL and Cry1Ac::ASAL fusion proteins, separately, exhibited receptor protein binding patterns of ~55 kDa & ~40 kDa, and ~70 kDa, ~55 kDa & ~40 kDa, respectively, after treatment with ASAL antibodies (Fig. 6). The present results suggest the existence of an additional binding site for the Cry1Ac::ASAL in BPH gut cells (Fig. 6). Earlier it was shown that the replacement of loops in Cry1Ab domain II resulted in the identification of additional binding sites^58^. Modification of Cyt2Aa toxin by the addition of peptides caused higher toxicity against pea aphids as compared to the replacement of loops with the peptides^59^.

The major objective of the present study has been to custommake rice plants using a novel *cry1Ac::asal* fusion gene which encodes a potent insecticidal protein having the ability to bind to different receptors of the insect gut cells and bestow enhanced toxicity as well as durable resistance against an array of insect pests. The Cry1Ac::ASAL fusion protein containing domains of both bacterial (Cry) and plant (ASAL) origin would be helpful in delaying insect resistance besides minimizing pest populations. The increased entomotoxic effects of fusion protein against lepidopteran and hemipteran insects are attributable to its higher binding affinity towards more number of receptor proteins in the insect gut epithelial cells. An overview of the present results demonstrates that the presence of ASAL in combination with two domains of Cry1Ac culminates in accentuated toxicity of the fusion protein to yellow stem borer, leaf folder and brown planthopper insects of rice. Accordingly, the newly designed fusion protein holds promise and may be deployed as a potent toxin against major lepidopteran and sucking pests of various other crop plants.

## Materials and Methods

### Development of *cry1Ac::asal* fusion gene construct for rice transformation

Earlier, a fusion gene *cry1Ac::asal* was constructed in our laboratory containing DI and DII domains (1089 bp) of Cry1Ac and a 375 bp fragment encoding carbohydrate binding domain of ASAL^20^. The *cry1Ac::asal* fusion gene of 1464 bp was amplified by PCR using 5′-GGCCATGGAGTTCGCCAGGAACAAGG-3′ and 5′-CCCGGGTCAACCCACACTTCT TCTGTAGG-3′ as forward and reverse primers employing pET28 vector as a template. Primers used for amplification contained the restriction sites *Nco* I and *Sma* I for cloning into the plant expression vector, and amplified PCR product was cloned at *Sma* I site of pBSK(+) plasmid. The recombinant clone was confirmed by restriction analysis and DNA sequencing method. Later, the fusion gene was excised using *Nco* I and *Sma* I restriction enzymes and cloned into pRT100 vector between CAMV 35 S promoter and *polyA* terminator. Finally, the fusion gene cassette was excised and cloned at *Hind* III site in pCAMBIA3300 vector containing *bar* gene as a selectable marker. The resultant pCAMBIA3300-CaMV35S-*bar*-CAMV-*cry1Ac::asal* vector was mobilized into *Agrobacterium* (EHA105) by tri-parental mating using helper plasmid PRK2013. Plasmid DNA was isolated from EHA105 cells and digested with *Hind* III enzyme to confirm the presence of the gene cassette.

### *Agrobacterium*-mediated genetic transformation and development of transgenic plants

Seeds of rice cultivar Pusa Basmathi (PB1), obtained from the Indian Institute of Rice Research (IIRR), Hyderabad, were used for rice transformation. *Agrobacterium*-mediated genetic transformation experiments were performed using EHA105 harbouring pCAMBIA3300-CaMV35S-*bar*-CAMV-*cry1Ac::asal* vector^60^. For selection of transformants, the co-cultivated calli were subjected to two rounds of selection on medium containing phosphinothricin (6 mg/l and 8 mg/L) for 2 weeks each. Later, the actively proliferating calli were selected and transferred onto regeneration medium^60^. Regenerated shoots were transferred to the rooting medium, and the rooted plants were transferred to pots. All the regenerated plants were grown to maturity in the glasshouse and putative transgenics were identified using herbicide (0.25%) Basta^61^.

### Molecular analysis of transgenic plants

Genomic DNA was isolated from the putative transformants and untransformed control (UC) plants^62^. For PCR analysis, primers 5-′GAATTCGAGTTCGCCAGGAACCAG-3′ & 5′-GGATCCGATGATGCTCACGGAACTG-3′ for *cry1Ac* gene, and 5′-GGATCCGCTATTCTAACCATACTG-3′ & 5′-GAGCTCACCCACA CTTCTTCTGTAGG-3′ for *asal* gene, 5′-CTACCATGAGCCCAGAACG-3′ & 5′-TCAG ATCTCGGTGACGGG-3′ for *bar* gene, and 5′GCTCAACACATGAGCGAAAC-3′ *polyA* reverse primer were used. Later, the PCR products were separated on 1% agarose gel and analyzed.

For Southern blot analysis, 15 g of genomic DNA samples from transformants as well as UC plants were digested separately with *Hind* III and *Eco*RI enzymes. The digested DNAs were electrophoresed on a 0.8% agarose gel and subsequently transferred to N+ Nylon membranes (Amersham Biosciences), and were fixed by exposing to UV (1200 J for 60 s) in an UV cross-linker. The membranes were separately probed with *cry1Ac::asal* and *bar* coding sequences labeled with α-^32^P dCTP, employing ready-to-go random primer DNA labelling kit (Amersham Biosciences). Further, the processing of membranes was done according to the manufacturer’s instructions.

### RT-PCR analysis

Total RNA was isolated from transgenic and UC plants using the TRIZOL method (Invitrogen, Carlsbad, CA, USA). The first strand cDNA generated was used as a template along with the primers 5′-GGCCATGGAGTTCGCCAGGAACAAGG-3′ & 5′-CCCGGGTCAACCC ACACTTCTTCTGTAGG-3′ for fusion gene (*cry1Ac::asal*), and 5′-GGATCCGCTA TTCTAACCATACTG-3′ & 5′GCTCAACACATGAGCGAAAC-3 for *asal-polyA*, to detect the presence of corresponding gene transcripts in the transgenic plants. Amplified products were analyzed by the gel electrophoresis on 1.0% agarose gel.

### Western blot analysis of transgenic rice plants

Samples of transgenic and UC plants’ leaf tissue were homogenized in 50 mM Tris-HCl buffer (pH 9.0). The extracts were centrifuged at 5,000 g for 20 min at 4 °C, and supernatants were collected and the protein samples (5 μg) were subjected to 15% SDS-PAGE^63^. Subsequent to electrophoresis, the separated proteins were transferred onto nitrocellulose N-membrane (Amersham) by electroblotting^64^. After protein transferring, the membrane was blocked by incubating in PBS solution containing 10% non-fat dried milk and 0.1% Tween 20 for 2 h at room temperature. Later, the membrane was probed with polyclonal rabbit anti-ASAL serum (1:10,000 dilution) as primary and goat anti-rabbit IgG horse-radish peroxidase conjugate (GENEi) as secondary antibody (1:10,000 dilution). The membrane was washed and treated with saturated benzidine solution containing 20% ammonium chloride and 0.1% H_2_O_2_.

### ELISA analysis

The microtitre plate wells were coated with 1 μg of crude protein extract of transgenic and UC rice plants and were kept overnight at 4 °C. The wells were washed thrice with 20 mM PBS containing 0.05% Tween 20 and were blocked with 10% non-fat dried milk for 2 h at 37 °C; later they were washed six times with PBS-T. The primary antibody of Cry1Ac (1:10,000) was added to the wells and incubated for 2 h at 4 °C. After incubation, the wells were washed thrice with PBS and incubated with secondary antibody, goat anti-rabbit IgG horse-radish peroxidase conjugate (GENEi) (1:10,000) for 1 h at room temperature. The plates were washed thrice with PBS, and 0.001% 3,3′,5,5′- tetramethylbenzidine (TMB) substrate in 0.05 M phosphate citrate buffer was added along with 0.1% H_2_O_2_ and kept in dark for 10 min. The reaction was stopped by adding 1 N H_2_SO_4_ and the absorbance was recorded on ELISA reader at 450 nm.

### Insect bioassays

The brown planthopper, leaf folder, and yellow stem borer were maintained on 25 to 30-day-old Taichung Native 1 (TN1) plants under controlled conditions. The freshly hatched nymphs or the nymphs after attaining the desired age were utilized for various experiments. For insect bioassays, six-week-old transgenic rice plants of PB-F_1D_, PB-F_3D_, PB-F_4D_, PB-F_8D_, PB-F_17D_, and PB-F_20A_ expressing the Cry1Ac::ASAL toxin were used and each experiment was repeated three times.

### Bioassays against leaf folder (LF)

The length of each individual second instar larvae of leaf folder was measured and allowed to starve for 5 h. Later, they were placed onto leaves of transgenic and UC plants (kept in Petri plates containing moist filter papers) and were incubated at 25 ± 2 °C in dark at 70% relative humidity for three days. After three days, insect mortality and leaf area damage were recorded. Percentage of leaf area damage was calculated using the formula: % leaf area damage = consumed leaf area/total leaf area before bioassay × 100.

### Cut-stem bioassays against yellow stem-borer (YSB)

Egg masses of yellow stem borer were collected from the rice field and allowed to hatch in the laboratory. Fresh stems of transgenic and control plants were harvested at the tillering stage and were cut into 5–6 cm long pieces. Twelve first-instar larvae of yellow stem borers and five pieces of freshly cut stems from each plant were placed in a sealed glass bottle and were incubated at 25 ± 2 °C in dark at 70% relative humidity for five days. After 5 days, the mortality rate of the insects was recorded.

### Bioassay with brown planthopper (BPH)

Thirty day old homozygous transgenic rice plants and UC plants were used to assess insect mortality/survival using no choice method. Early first instar nymphs of BPH (20 each) were released on each plant that was confined in an insect proof mylar cage. Nymphal survival was monitored and observations were taken at 3 day intervals up to 24 days under controlled environmental conditions (25 ± 2 °C and 70% relative humidity). The delay in the development of insects was also observed daily by scoring the number of adults and insects still in nymphal stage.

The effect of fusion protein on the fecundity of insects was estimated by scoring the nymphs emerged from the hatched eggs. For this study, surviving male and the female insects were pooled and confined again in a 1: 1 ratio, so that there is no difference in the nymph production based on the sex ratio. The nymphs emerging from a pair of adults were counted daily up to 7 days, after which surviving adults were removed and the plants were observed for unhatched eggs by adopting the staining technique^1^. A sum of the emerged nymphs and unhatched eggs were used for estimating the fecundity. Plant damage was assessed visually and compared with susceptible TN1 rice plants (100% damage).

### Semi-quantitative assay of honey dew production (feeding behavior) of BPH

The extent of insect feeding was estimated by semi-quantitative honeydew assay (liquid excreta) produced by the insects. Whatman No.1 filter paper, dipped in bromocresol green solution (2 mg/ml in ethanol) was placed at the base of each plant and covered with a plastic cup. On each plant, five adult female BPH insects, pre-starved for two hours, were released separately and allowed to feed for 24 h. Care was taken not to release gravid adult females. Honeydew, excreted by the insects, reacts with bromocresol green on the filter paper resulting in blue colour. The area of blue spots developed on filter papers were measured using millimeter graph paper and expressed in units (1 unit = 1mm^2^)^1^.

### Western blot analysis to detect insect receptor proteins binding to the fusion protein

Insects fed on transgenic rice lines and untransformed control plant were collected and homogenized in 50 mM Tris-HCl buffer (pH 9.0) to isolate total proteins of the insects. The extract was centrifuged at 5,000 g for 20 min at 4 °C, and the supernatant was collected and the protein samples (5 μg) were subjected to 15% SDS-PAGE^63^. The separated proteins were transferred onto nitrocellulose N- membrane (Amersham) by electroblotting^64^. After protein transfer, the membrane was blocked by incubating in PBS solution containing 10% non-fat dried milk and 0.1% Tween 20 for 2 h at room temperature. The membrane strips were probed with polyclonal rabbit anti-ASAL serum (1:10,000 dilution), followed by goat anti-rabbit IgG horse-radish peroxidase conjugate (GENEi) as a secondary antibody (1:10,000 dilution). Membrane strips were washed and treated with saturated benzidine solution containing 20% ammonium chloride and 0.1% H_2_O_2_.

## Electronic supplementary material

Supplementary Information
