# Supplementary material for: Expression of hybrid fusion protein (Cry1Ac::ASAL) in transgenic rice plants imparts resistance against multiple insect pests
Source: Sci Rep. 2018 May 31;8:8458. doi: 10.1038/s41598-018-26881-9 (PMC5981619; doi:10.1038/s41598-018-26881-9)
Supplement: Supplementary file 1 — Supplementary Information [file 41598_2018_26881_MOESM1_ESM.pdf]

Expression of hybrid fusion protein (Cry1Ac::ASAL) in transgenic rice plants imparts resistance against multiple insect pests

Dayakar Boddupally, Srinath Tamirisa, Sivakrishna Rao Gundra, Dashavantha Reddy Vudem and Venkateswara Rao Khareedu*

Centre for Plant Molecular Biology, Osmania University, Hyderabad, 500 007, India

*Corresponding author:

Prof. Khareedu Venkateswara Rao

Centre for Plant Molecular Biology

Osmania University

Hyderabad, 500007, India

Telephone: 91-40-27098087

Fax: 91-40-27096170

E-mail:rao_kv1@rediffmail.com

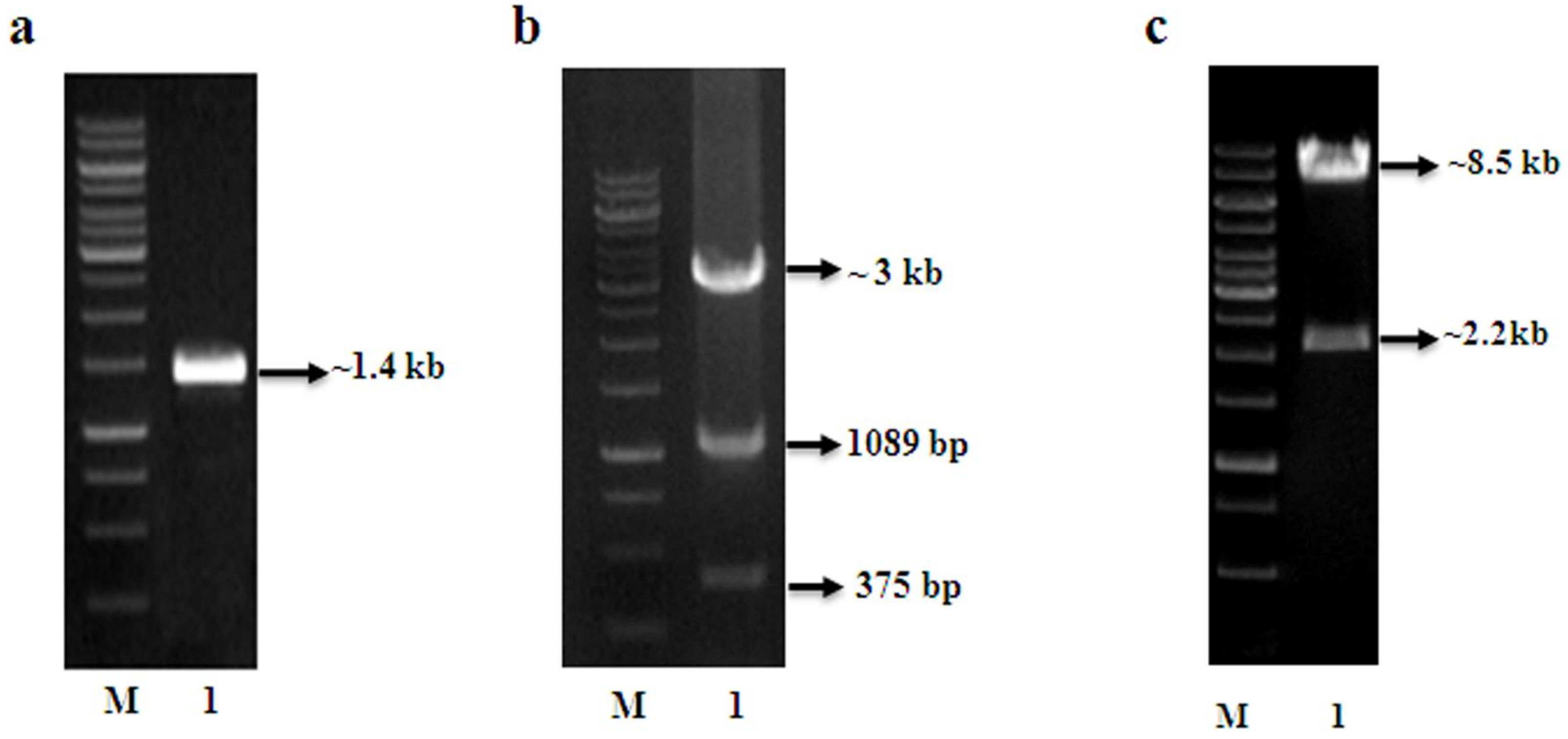

Supplementary Figure. S1. Amplification of *cry1Ac::asal* fusion gene and cloning of fusion gene in pCAMBIA3300 plant transformation vector. **(a)** Amplification fusion gene by PCR using sequence specific primers. M: 1kb DNA Ladder; Lane 1: Amplified fusion gene. **(b)** Cloning of fusion gene into pRT100 vector. Restriction analysis of pRT100 vector carrying *cry1Ac::asal* fusion gene. M: 1 kb DNA Ladder; Lane 1: *Nco* I and *Bam* HI digestion of pRT100 *cry1Ac::asal* fusion gene vector. **(c)** Restriction analysis of pCAMBIA3300 plant transformation vector. M: 1 kb DNA Ladder; Lane 1:pCAMBIA3300 recombinant vector digested with *Hind* III restriction enzyme.

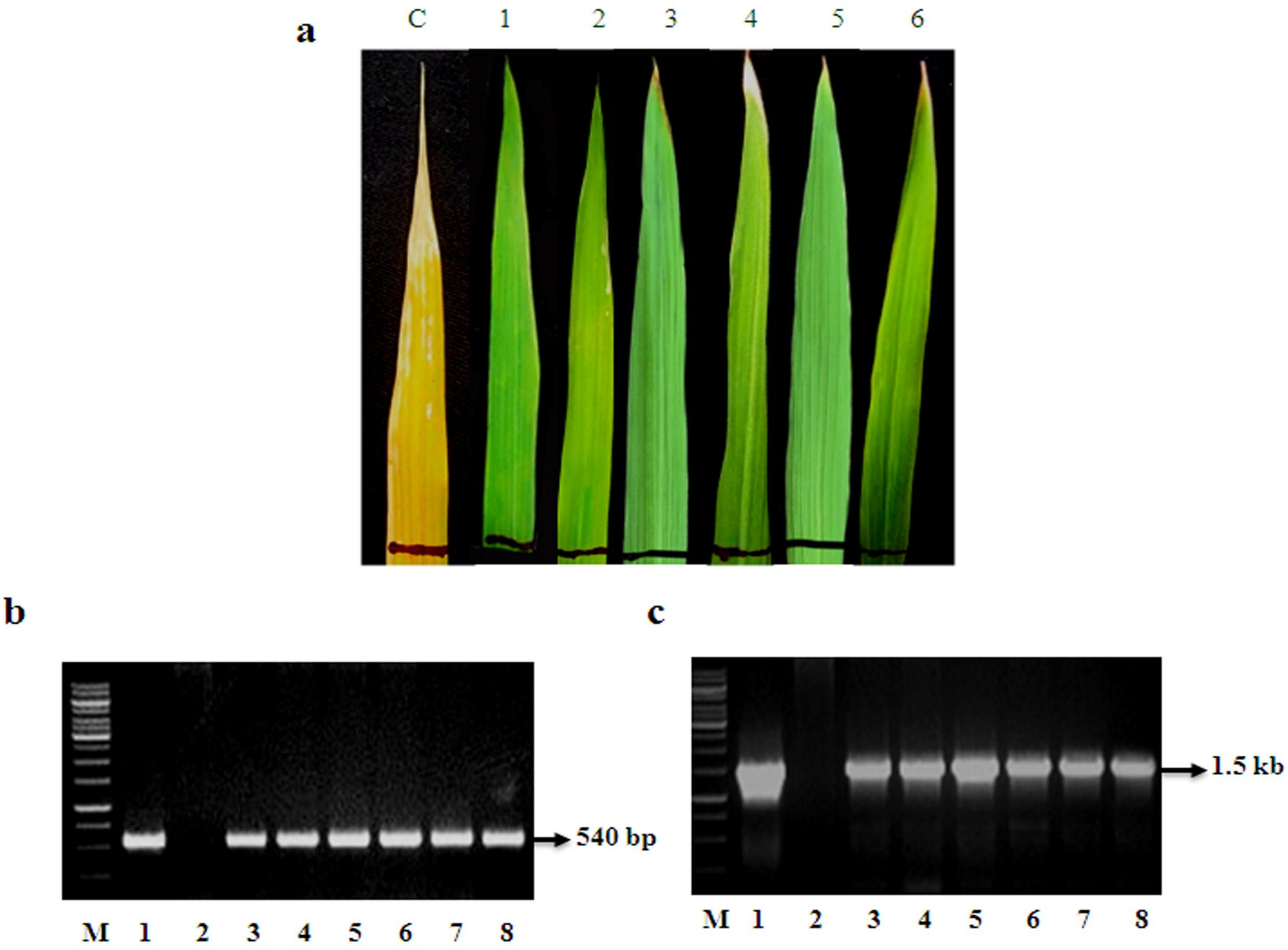

Supplementary Figure. S2. Confirmation of putative transgenic lines using Basta leaf dip assay and PCR analysis. **(a)** Putative *cry1Ac::asal* transgenic rice leaves treated with herbicide Basta showing varied levels of tolerance. Lane C: represents untransformed control plant showing complete damage. Lanes 1, 2, 3, 4, 5 & 6. Different *cry1Ac::asal* transformants showing herbicide tolerance. **(b)** PCR analysis of *cry1Ac::asal* transgenic plants using *bar* gene specific primers. Lane M: 1.0 kb marker. Lane 1: Positive control. Lane 2: Untransformed control. Lanes 3, 4, 5, 6, 7 & 8: Different *cry1Ac::asal* transgenic lines. **(c)** PCR analysis of *cry1Ac::asal* transgenic plants using *cry1Ac::asal* fusion gene specific primers. Lane M: 1.0 kb marker. Lane 1: Positive control. Lane 2: Untransformed control. Lanes 3, 4, 5, 6, 7 & 8: Different *cry1Ac::asal* transgenic lines.
